# Assessment of a markerless motion analysis system for manual wheelchair application

**DOI:** 10.1186/s12984-018-0444-1

**Published:** 2018-11-06

**Authors:** Jacob Rammer, Brooke Slavens, Joseph Krzak, Jack Winters, Susan Riedel, Gerald Harris

**Affiliations:** 1 Orthopaedic and Rehabilitation Engineering Center (OREC), Marquette University, Olin Engineering Suite 323, Milwaukee, WI 53201-1881 USA; 2 Department of Biomedical Engineering, Marquette University, Olin Engineering Suite 323, Milwaukee, WI 53201-1881 USA; 3 Department of Orthopaedic Surgery, Medical College of Wisconsin, Milwaukee, WI 53201-1881 USA; 4 Shriners Hospitals for Children, Chicago, IL USA; 5 Midwestern University, Physical Therapy Program, 555 31st St., Alumni Hall 340C, Downers Grove, IL 60515 USA; 6 University of Wisconsin-Milwaukee, 2400 E Hartford Ave, Rm. 983, Milwaukee, WI 53211 USA; 7 Marquette University, Biomedical Engineering, Milwaukee, WI 53201-1881 USA

**Keywords:** Manual wheelchair, Markerless motion capture, Musculoskeletal models, Pediatric rehabilitation

## Abstract

**Background:**

Wheelchair biomechanics research advances accessibility and clinical care for manual wheelchair users. Standardized outcome assessments are vital tools for tracking progress, but there is a strong need for more quantitative methods. A system offering kinematic, quantitative detection, with the ease of use of a standardized outcome assessment, would be optimal for repeated, longitudinal assessment of manual wheelchair users’ therapeutic progress, but has yet to be offered.

**Results:**

This work evaluates a markerless motion analysis system for manual wheelchair mobility in clinical, community, and home settings. This system includes Microsoft® Kinect® 2.0 sensors, OpenSim musculoskeletal modeling, and an automated detection, processing, and training interface. The system is designed to be cost-effective, easily used by caregivers, and capable of detecting key kinematic metrics involved in manual wheelchair propulsion. The primary technical advancements in this research are the software components necessary to detect and process the upper extremity kinematics during manual wheelchair propulsion, along with integration of the components into a complete system. The study defines and evaluates an adaptable systems methodology for processing kinematic data using motion capture technology and open-source musculoskeletal models to assess wheelchair propulsion pattern and biomechanics, and characterizes its accuracy, sensitivity and repeatability. Inter-trial repeatability of spatiotemporal parameters, joint range of motion, and musculotendon excursion were all found to be significantly correlated (*p* < 0.05).

**Conclusions:**

The system is recommended for use in clinical settings for frequent wheelchair propulsion assessment, provided the limitations in precision are considered. The motion capture-model software bridge methodology could be applied in the future to any motion-capture system or specific application, broadening access to detailed kinematics while reducing assessment time and cost.

## Introduction

There are several current methods that have been successfully applied to study certain aspects of wheelchair propulsion outcomes and biomechanics. Laboratory motion analysis [1–3] is precise and detailed, yet costly and time-consuming, especially for repeated, frequent longitudinal assessments. Inertial Measurement Units (IMUs) are easier to use, but, when wrist-applied as typical practice [4] lack detailed joint kinematics at the shoulder, a key joint in assessing injury risk for manual wheelchair users. Leving et al. [5] has shown promise for IMUs to detect spatiotemporal parameters of motion, including activity level, but does not describe movement kinematics. Similarly, van der Slikke et al. [6–9] have shown accurate measurements by IMUs in speed, displacement, and acceleration metrics applied to wheelchair sports. The referenced IMU studies do not address the kinematics of individual joints, rather focusing on gross movement of the wheelchair and spatiotemporal aspects of arm movement for propulsion. Instrumented wheels, whether commercially available like the SmartWheel [10] or modified from bicycle wheel power meters [11] provide power and torque output at the pushrim, but require a motion capture system to obtain kinematics. Standardized outcome measures like the Wheelchair Propulsion Test (WPT) and Wheelchair Skills Test (WST) use trained observers and standard protocols to assess function [12], but lack quantitative, kinematic data.

Based on the available solutions on the market, there is a significant need for development in this area, targeted toward physical and occupational therapists. The proposed technology quantitatively evaluates manual wheelchair mobility in a timely manner and outside of the motion analysis laboratory. The system output includes spatiotemporal parameters, joint and muscle kinematics, and propulsion pattern. The spatiotemporal parameters, which can tell the clinician how propulsion speed, cadence, and time change during a patient’s rehabilitation process, indicate efficiency to the clinician. It also includes joint range of motion, which is useful in determining which joints are being utilized, and whether the patient is progressing toward a more effective strategy, Propulsion pattern is additionally provided as a qualitative (pattern) and quantitative (size of the pattern) metric – useful for assessing changes in response to therapy, and progress toward smoother and more effective propulsion. This monitoring could provide clinicians with quantitative data to indicate whether a patient is stable or deviating from an appropriate pattern during the course of care. This has a potential to be an early indicator of injury risk, as cadence and propulsion pattern were identified as predictors of injury risk in manual wheelchair users with spinal cord injury [13]. Several technological options have recently become available to make this development possible, including the Kinect® for motion capture, OpenSim for musculoskeletal biomechanics, and the Personal Wheelchair Platform to support the wheelchair and simulate overground resistance. Each technological element of the system will be introduced and discussed separately.

### Microsoft® Kinect®

The Microsoft® Kinect® is a markerless motion capture sensor designed and marketed for the consumer gaming market. It uses infrared depth sensing to capture 3-dimensional imaging and real-time algorithms to process skeletal position. The validity and research applicability of the Kinect has been widely debated in current literature, as summarized in Table 1. Several studies ([14–17], and [18]) have compared the Kinect against motion capture systems and have reported that the Kinect-detected data is reproducible, accurate for gross movement detection but not finer movements, and approximately one order of magnitude lower precision than the laboratory standard marker-based systems. Studies focusing on specific aspects of detection found that shoulder kinematics and range of motion are reliable [19, 20] and test-retest reliability is acceptable in both healthy and stroke patients [21]. Based on these findings, we focus on shoulder biomechanics in our analysis.

**Table 1:** Microsoft Kinect in Upper Extremity Clinical Applications [49–68]

Reference	Description	Key Results
[14]	Assessment of validity of Kinect v1.0 against marker-based motion capture; 48 normal subjects; upper and lower extremity	Similar reproducibility; different ROM detection for the lower extremity but similar results for shoulder abduction (±3°) and elbow flexion (±11°)
([45, 46]; [47, 48])	Assessment of validity of Kinect v2 for postural control and balance against marker-based motion capture; 30 normal subjects;	High reliability and concurrent validity for balance assessment (trunk, upper and lower extremity kinematics)
[15]	Direct comparison of Kinect against Vicon ® clinical motion capture	Kinect detection is accurate, one order of magnitude less precise than Vicon
[16]	Kinect vs. Vicon for gross and fine movements (controlled study of Parkinson’s disease); movements included whole-body coordinated movements and shoulder flexion/abduction targeted movements	Kinect is highly accurate for gross movement detection, less for smaller hand movements; repeatable measurements (*r* > 0.9); high interclass correlation for gross extremity/body movements; low correlation for fine hand movements
[19]	Shoulder-specific validity and reliability of Kinect; 10 normal subjects; shoulder joint (flexion, abduction, rotation) assessed in static poses with Kinect, marker based motion analysis, and goniometer; the Kinect was tested both in anterior and sagittal view with insignificant difference in ICC	High reliability, but LOA greater than ±5°, up to 7° for shoulder abduction; Kinect shoulder measurement is most accurate in flexion (high ICC with valid measurements), and least accurate at abduction approaching 90°; note that the analysis focused on extents of motion, not the entire range of motion
[20]	Shoulder ROM by Kinect vs. goniometry; 15 normal and 12 with adhesive capsulitis of the shoulder; Active ROM compared between standard goniometry and Kinect	High ICC; Kinect is repeatable for shoulder ROM measurements (ICCs: 0.91 flexion, 0.94 abduction; 0.91 external rotation); Kinect accurately measures 3D shoulder ROM
[21]	Test-retest repeatability of Kinect for UE, both 12 healthy and 18 stroke subjects; focus on shoulder and elbow kinematics, and spatiotemporal metrics	Study showed acceptable repeatability and sensitivity in both populations; Shoulder and elbow angle measurements all showed greater than 0.9 ICC, indicating repeatability
[17]	Accuracy and reliability of Kinect v2 for clinical measurements – compared with Vicon; 19 normal subjects; spatial range of motion of arm movements evaluated	Most parameters ICC > 0.7; no systematic bias; all joints of the UE and torso detected by Kinect had Pearson correlation > 0.9 against Vicon; concurrent Kinect and Vicon used
[18]	Kinect (anterior) vs. Vicon; 20 normal subjects; balance and arm sway; Kinect and Vicon data collected separately, analyzed for variance in movement patterns and marker positions	Study found that broad movements of the upper extremities had > 90% accuracy, finer hand movements lower accuracy; activities are standardized (game-directed) for comparison between the systems

*ICC* Interclass Correlation Coefficient, *ROM* Range of Motion, *LOA* Limits of Agreement; most studies use Kinect in anterior position, noted if different

Specifically focusing on the elbow and shoulder movements most relevant to manual wheelchair propulsion, several studies have addressed accuracy and reliability of the Kinect for this use. Comparing the shoulder kinematics from Kinect to laboratory motion capture, Bonnechere et al. found that ROM detection is within 3 degrees for shoulder abduction and 11 degrees for the elbow, with the Kinect sensor positioned anterior to the subjects. Huber et al., addressed all ranges of shoulder movement in three axes, and found that the Kinect is most valid in flexion (throughout the range of motion), with an ICC of 0.95 when compared to laboratory calibrated measures, and least accurate in extreme abduction approaching 90 degrees, with ICC of 0.76. Overall analysis of these results in terms of minimum detectable difference demonstrates differences by joint motion, with 7° at the shoulder [14], and 11° at the elbow [19]. However, these results also show that these measures are repeatable with high correlation coefficients [20], which suggests that the data that ultimately is processed from the Kinect is able to detect kinematic changes, even if the measurement accuracy is less than laboratory-grade systems.

In terms of manual wheelchair propulsion, the most important movement of the shoulder joint is in flexion, and there is no extreme abduction, so these results suggest that the Kinect is adequate in the ranges of motion applicable to manual wheelchair use. Huber et al. also compared shoulder flexion with the Kinect positioned anteriorly and laterally, and found similar ICC (0.85 and 0.84, respectively) between the positions. This provides a basis for the experimental assessment contained in this work, assessing detection accuracy within the specific workspace of manual wheelchair use and camera positioning applied.

In past work [22], technical evaluation of the system using goniometry revealed key findings regarding the capabilities of the system. The broad movements of the elbow demonstrate more precision in detection than the finer movements of the hand, a result expected due to the limited resolution of the Kinect. Detection accuracy when comparing Kinect-detected and goniometric measurements is significant enough to allow differentiation between angles of the joints, and provides sufficient kinematic data for clinical decision-making. Overall, this work indicates that the Kinect is accurate in detecting ROM and joint position of the upper extremities, with a reduced precision of approximately one order of magnitude relative to laboratory systems, and higher accuracy and precision in the proximal joints relative to the distal joints. For the purposes of this development, the Kinect adequately provides the desired level of quantitative data, but the Kinect’s limitations must be accounted for when interpreting that data.

### OpenSim musculoskeletal model

OpenSim is a free, open-source software package that allows users to develop musculoskeletal models and perform biomechanical analysis [23]. The OpenSim software (National Center for Simulation in Rehabilitation Research) and specific upper extremity model used were chosen over other alternatives (including SIMM, Any-Body, and other OpenSim models) in line with the primary project goals of cost-effectiveness, research validity and acceptance in the literature, and integration into assessment software. OpenSim has gained a significant following in scientific literature, with many studies published using the software. OpenSim is also computationally efficient, while providing sufficient data to be appropriate for this application. Given that the system is open-source, it is also easily integrated into the automated assessment software. Several upper extremity models are available that are applicable to the study of wheelchair propulsion biomechanics. Holzbaur et al. [24] developed a complete model designed to accurately represent musculoskeletal structure. The validated model was later refined [25] and enhanced for improved functionality. The newer model also incorporates scapular kinematics, and a simplified coordinate system for enhanced computational efficiency, and thus was applied for this work.

### Stationary wheelchair propulsion platform

Roller platforms and similar ergometer devices are often used in wheelchair propulsion research, placing the wheelchair in a fixed position during analysis. This is important because it allows the wheelchair user to reach a steady-state propulsion. Some laboratories [13, 26] develop research-specific systems tailored to their needs. For instance, van der Woude et al. [2] describe a custom-developed motor-driven treadmill combined with a weight-and-pulley system to provide resistance, which they use in parallel with motion capture, energetics, and instrumented wheels. Recent development has led to the Personal Wheelchair Platform [27], which provides a safe, stable, laterally independent, and calibrated platform for manual wheelchair propulsion research. The platform is designed based on a dynamic model to provide resistance consistent with overground propulsion, and does not control or limit testing conditions. The design is entirely mechanical and, if used with the same wheelchair, would produce the same results.

## Methods

**Fig. 1:**
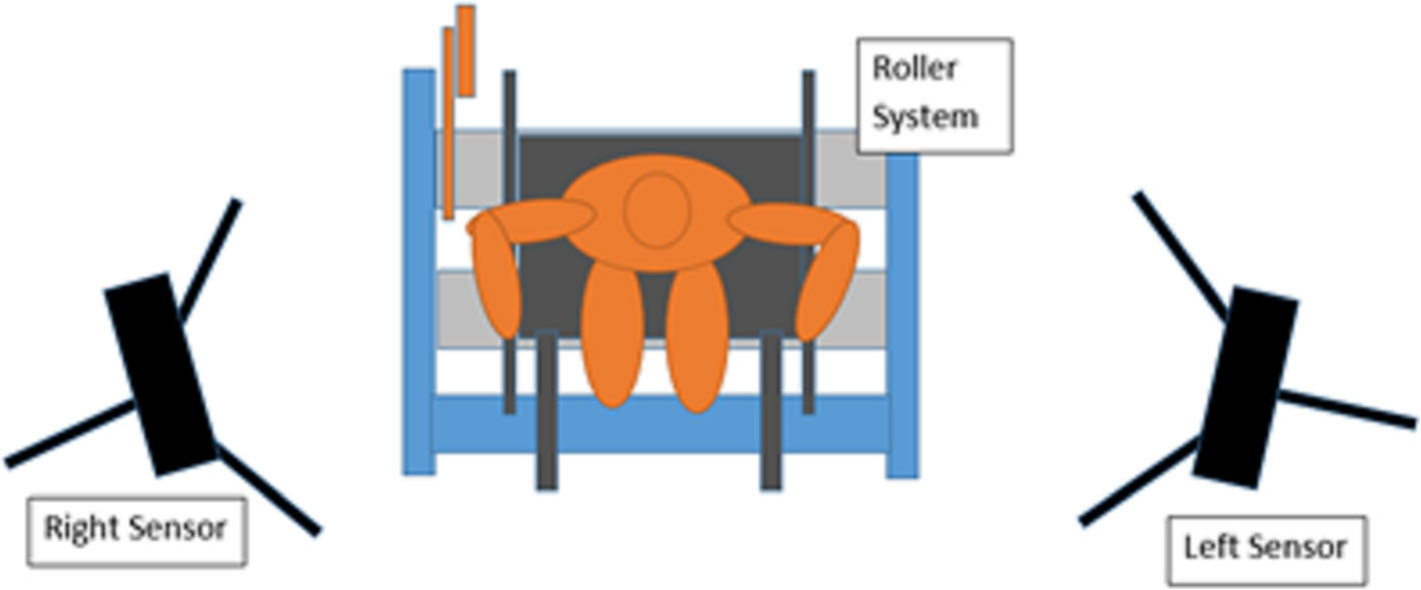
Conceptual Design and Configuration of the Markerless Wheelchair Analysis System. Subject is stationary on roller system, with a single Kinect sensor positioned in the center, anterior to the subject (for static trial), and two Kinect sensors positioned laterally, to the left and right of the subject (for dynamic trials) – the sensors are moved between trials and a total of two are needed

The purpose of this study was to develop and evaluate a markerless wheelchair propulsion biomechanical assessment system based on the actual needs of clinicians and wheelchair users, focused on shoulder and upper extremity kinematics. The resulting design integrates consumer technology with open-source musculoskeletal modeling technology, considering the important value and technical limitations of each component, to produce a markerless wheelchair propulsion analysis platform. The system was designed around three components: the Microsoft Kinect sensor, a stationary roller platform, and OpenSim musculoskeletal model. The system was configured with the subject and wheelchair in a stationary position on a roller platform, with Microsoft Kinect sensors placed anteriorly (for recording the static trial) and laterally on each side (for recording dynamic trials), as illustrated in Fig. 1.

The system needs two Kinect sensors for minimum operation. The Kinect produces the clearest tracking results when the primary motion is perpendicular to the sensor’s line of sight. Thus, for the static trial an anterior positioned sensor is used to detect the subject in standard anatomical position, while for dynamic trials the lateral cameras are used, since sagittal plane motion is the primary action of wheelchair propulsion. The laterally-positioned Kinects also minimize occlusion of wheelchair components and body parts, allowing the sensors to maintain their view of all upper extremity segments throughout the propulsion cycle.

**Fig. 2:**
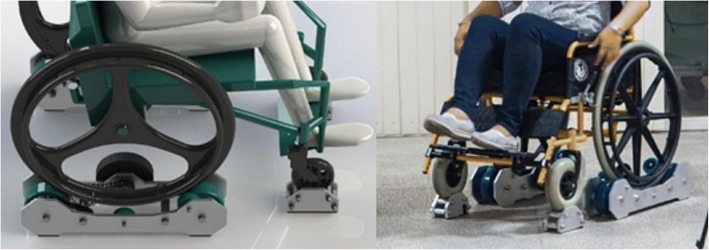
Personal Wheelchair Platform. Used to support the wheelchair and provide anthropometrically correct resistance

The Personal Wheelchair Platform (Fig. 2) supports the wheelchair, constrains its lateral motion, allows asymmetric propulsion, and provides adjustable resistance to simulate overground propulsion based on user anthropometry. Maintaining the wheelchair in a static position is key to using the markerless technology effectively. Maintaining the static position allows consistent accuracy of the kinematics and, most importantly, allows the subject to continually propel forward rather than making repeated turns within a laboratory setting. This supports community and home applications.

The final major component of the system is an OpenSim-based musculoskeletal model, developed and validated for upper extremity kinematics [25]. The model employed a virtual marker set compatible with the automated algorithms that interpret data from the Kinect sensors. The model is iteratively fitted to the motion data, and to increase the simplicity and speed of the computations, the model is used in its unilateral configuration, with each upper extremity computed separately. Key kinematic data outputs from the model include triaxial joint kinematics of the arms and trunk, and musculotendon lengths.

**Fig. 3:**
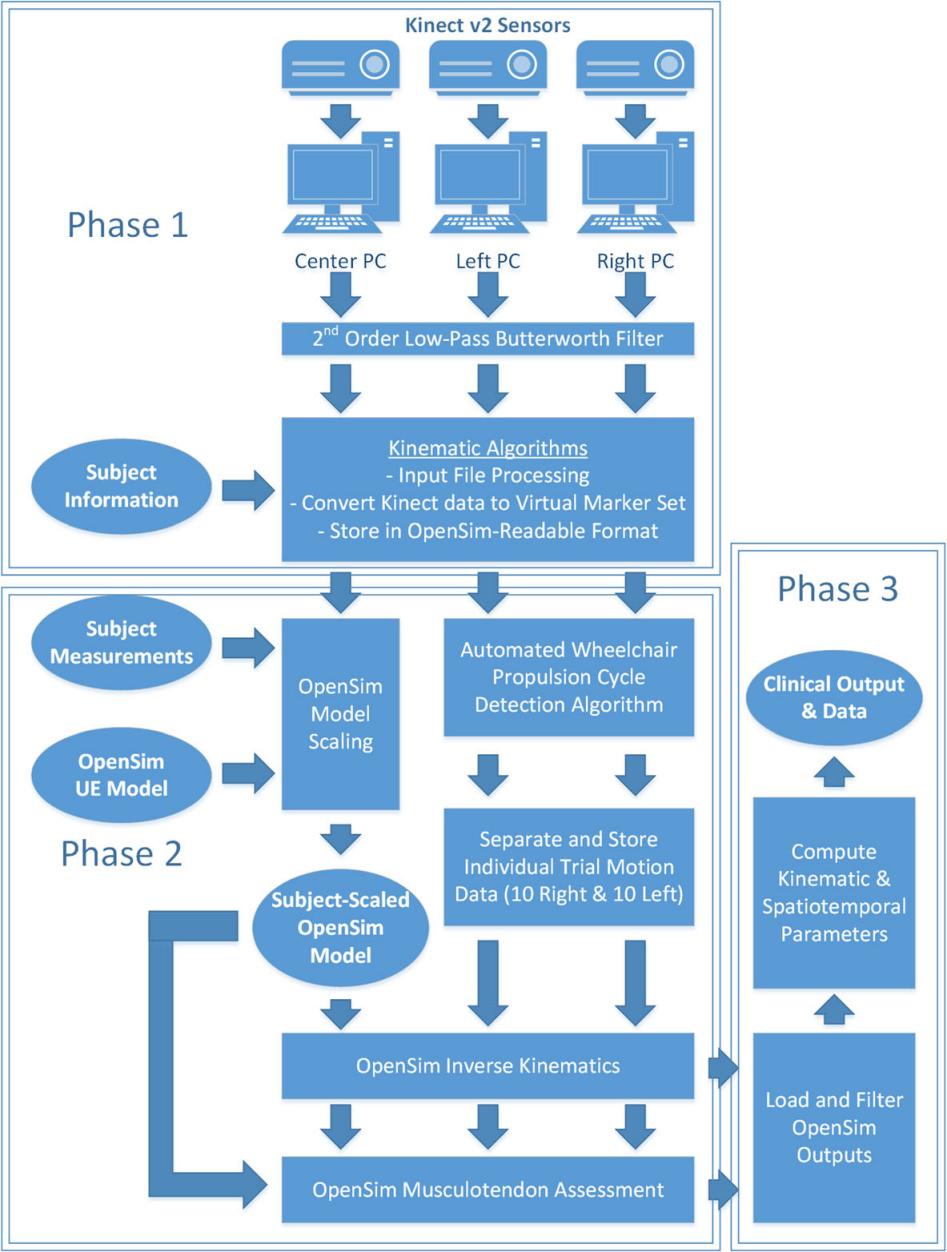
Block Diagram of Markerless Kinematic Processing Algorithm*.* Phases 1, 2, and 3 of processing referenced in text are denoted by boxed regions

The Microsoft Kinect produces skeletal position, which is recorded in real-time from the sensors during the evaluation, and is subsequently input to the OpenSim musculoskeletal model. The software package was developed using MATLAB, which can interface with both the Kinect software and OpenSim modeling package when appropriately configured. Figure 3 illustrates the process, and is divided into three distinct processing phases.

Phase 1 of the process imports and filters the skeletal position data, acquired from both static and dynamic trials. A low-pass Butterworth filter, with cutoff frequency of 10 Hz, removes unwanted noise from the position data. The OpenSim model has been modified to include a custom virtual marker set that interfaces with data acquired by the sensors. The final step in Phase 1 converts position data, consisting of joint center locations and segment quaternion orientations, to virtual marker positions.

Phase 2 then includes a second set of algorithms to process the position data, automatically identifying individual propulsions from the data series, and selecting the ten most similar and consistent propulsions from those identified. The data is then divided into twenty individual data sets comprised of ten propulsions each on the left and right sides and is ready for OpenSim processing. Each individual trial is processed separately, producing an individual set of kinematic data for each trial. OpenSim processing is conducted by customized MATLAB algorithms. First, the model is scaled using data from the static trial and measurements provided by the evaluator. The scaling is proportional and uses the anatomical scaling capability of OpenSim, adequate for gross kinematic analysis in clinical research. The joint kinematics and musculotendon lengths from the static trial are recorded as the baseline, normal values. Next, each dynamic trial is processed using the subject-specific model. The iterative inverse kinematics method fits the model to the motion data at each time point. Then, muscle analysis is conducted, using geometric mapping to compute the musculotendon length changes.

Phase 3 in Fig. 3 computes spatiotemporal parameters, joint ranges of motion, and musculotendon excursions, and average and standard deviation values for each parameter. A formatted output is created in MATLAB to display all relevant parameters and outputs of the evaluation. This output is displayed automatically on-screen and saved as an image file for printing. All raw and processed data and parameters are saved in a MATLAB archival data file for future research and processing.

**Fig. 4:**
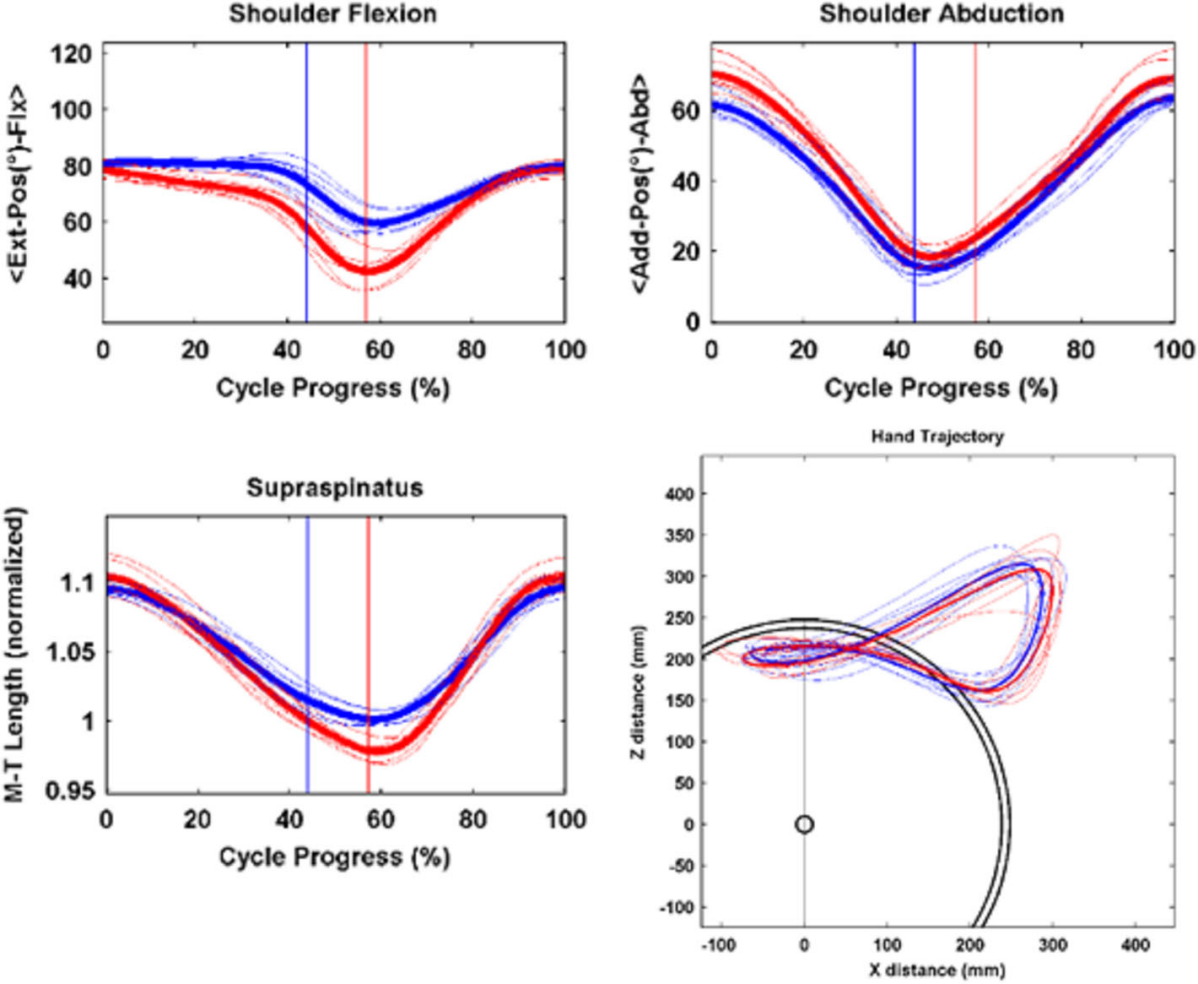
Example Clinical Outputs*.* Joint Kinematics and Spatiotemporal Parameters for exemplar subject, age 15, with spina bifida – Joint kinematics (top), musculotendon excursion (bottom, left) and propulsion pattern (bottom, right). The subject propelled using the same wheelchair and settings used for everyday mobility, at a self-selected speed and propulsion pattern. The thin lines represent individual trials, and thick lines are average of all trials for left and right extremities

The clinical wheelchair propulsion analysis output from the markerless system is two printable pages, created by the automated software. This format mimics reports produced for clinical gait analysis with marker-based systems and includes both kinematic plots and spatiotemporal parameter data in a standardized, easily interpreted format for clinical use. This work expands on the contributions to the field made by de Groot et al. [28]‘s WHEEL-I system and the OptiPush instrumented wheel, described in Kwarciak et al. [29]. Figure 4 gives an example of the kinematic outputs for a 15-year-old subject with spina bifida, provides kinematic plots of the joint motion of each key upper extremity joint and thoracic motion. Each plot of upper extremity joint motion presents the left (blue) and right (red) kinematics, with thin lines representing individual trials and thick lines representing the mean of all trials. The vertical blue and red lines on each plot indicate the point when the hand leaves the pushrim, which identifies the transition from propulsion phase to recovery phase. The first segment, from 0% to the vertical line, is the propulsion phase, where the hand is in contact with, and actively propelling, the pushrim. The second segment, from the vertical line to 100%, is the recovery phase, where the hand returns to its starting position. The transition between push and recovery phase is computed with an automated MATLAB script, which compares the hand’s position with the known semicircular arc of the pushrim at each time point, and is then able to estimate which data points have hand contact with the pushrim and which points are recovery phase. In the lower left corner of the first page, values are tabulated for range of motion, peak angular velocity, and peak angular acceleration of key joints. The values are averages across all trials, with left and right extremities presented separately.

To analyze the sensitivity of the model for the wheelchair propulsion task, sensitivity analysis was used to relate shoulder and elbow joint motion to musculotendon excursions for the muscles which cross the respective joints. The purpose of this analysis is to mathematically determine the effect of changing joint angle on musculotendon motion. This was performed by perturbing the model throughout the range of shoulder and elbow mobility expected in wheelchair propulsion and recording the musculotendon response length response for each measurement. Then, a linear regression model was used to determine the musculotendon sensitivity to joint motion. This method of using sensitivity analysis to assess the model performance is adapted from the formulas of Rump et al. [30], and while it has not yet been widely applied in the field of biomechanics, it has been a useful procedure in several other scientific fields, such as automotive injury prediction [31–35].

To more specifically address the sensitivity of the model at key points of interest in manual wheelchair propulsion, the shoulder joint is assessed at the start (hand contact) and end (hand release) of a typical wheelchair propulsion cycle, since these transition points represent the most significant potential for injury, as suggested by Rankin et al. [36], who found peak power output at the transition points. To set up the analysis, the model is fixed to the start and end points (based on typical values collected from subjects), and the other joints not being perturbed are fixed at those values. Thus, only the joint of interest is being perturbed for the sensitivity analysis. Analysis is performed using a dimensionless sensitivity coefficient. This is computed as:

((MTL + 5%)-(MTL -5%))/(Initial MTL)]/[((JA + 5%)-(JA-5%))/(Initial JA)

Where MTL = musculotendon length, and JA = joint angle.

Inter-trial measurement repeatability was analyzed using correlation analysis to provide scatterplots and Pearson correlation coefficients describing the repeatability of the measurement system, to test the hypothesis that the system reliably measures parameters between trials. Thirty wheelchair propulsion assessments of pediatric manual wheelchair users were conducted as part of an institutional review board-approved study. The subjects ranged from 6 to 17 years of age with 6 females and 24 males, 12 with spina bifida, 3 with Charcot-Marie-Tooth disorder, and 15 with cerebral palsy, all of whom use a manual wheelchair as a primary means of daily mobility.

The assessment included spatiotemporal parameters, such as push time, cycle time, frequency, contact angle, and proportion of push and recovery, similar to those used in Vegter et al. [37]. Additionally, joint range of motion and musculotendon excursion were analyzed, which differs from the kinetic analysis of Vegter. Each assessment was performed twice, and the two assessments are statistically compared – each subject’s data from the first assessment was compared to the same subject’s data from the second assessment. The subjects were instructed to perform the same manual propulsion task during each assessment. Speed and power output were results of the subject’s standard, self-selected pattern. A Lilliefors test of normality was performed on the differences between the data sets to ensure that the normality assumption of the parametric Pearson correlation analysis was satisfied.

## Results

### Application and kinematic results

The system developed during this project has been rigorously assessed in both laboratory and real-world applications. The cost-effectiveness, at approximately $8000 for the complete system, and simplified assessment protocol make the system viable in several key environments. Our pilot experience in field-testing the system suggested that assessments typically can be completed in under 15 min and training clinicians to use the system is feasible and can be accomplished in 30–45 min.

### Sensitivity analysis

Sensitivity analysis was performed on the musculoskeletal model to determine the relationship between joint motion and musculotendon excursion. For shoulder elevation, the anterior and posterior deltoid musculotendon complexes (− 0.08%/degree and 0.15%/degree, respectively), along with the coracobrachialis (− 0.09%/degree), have high sensitivity to shoulder elevation and rotation when compared to the other musculotendon complexes studied, mostly in the range of 0.01–0.03%/degree. Sensitivity analysis describing musculotendon response to shoulder elevation shows a clear transition to a point where the joint is not moving very much, but the muscles are quickly changing in length. The high linear velocity of the musculotendon complexes strongly suggests a high potential for injury during the wheelchair propulsion cycle.

Thus, each coefficient presented in Table 2 below is dimensionless, and the higher the coefficient, the more sensitive the muscle is to joint angle changes within the specified propulsion area. These coefficients are then categorized as moderately sensitive (0.40 < s < 0.75) or highly sensitive (s > 0.75).

**Table 2:** Sensitivity of Musculotendon Complexes to Shoulder Motion at Start and End Points of Propulsion

Muscle	Shoulder Elevation (Start Point)	Shoulder Elevation (End Point)	Shoulder Rotation (Start Point)	Shoulder Rotation (End Point)
Ant Deltoid	0.435*	−0.185	0.243	0.810**
Lat Deltoid	−1.409**	0.039	−0.779**	−0.175
Post Deltoid	−2.038**	0.288	−1.137**	−1.268**
Supraspinatus	0.118	−0.041	0.068	0.183
Infraspinatus	0.466*	−0.025	0.257	0.112
Subscapularis	−0.494*	0.028	−0.273	−0.124
Teres Minor	0.828**	0.038	0.456*	−0.169
Teres Major	1.493**	0.191	0.819**	−0.840**
Pectoralis Major	0.701*	−0.038	0.385	0.169
Latissimus Dorsi	1.369**	0.103	0.751**	−0.453*
Coracobrachialis	1.719**	−0.155	0.952**	0.683*
Triceps-Long	0.372	0.088	0.203	−0.385
Triceps-Medial	−0.287	−0.103	− 0.156	0.454*
Biceps-Long	0.778**	0.029	0.406*	−0.131
Biceps-Short	1.874**	0.008	1.010**	−0.037
Brachialis	0.318	0.077	0.172	−0.341

Values presented as dimensionless sensitivity coefficients with +/− 5% perturbation at the start and end points of propulsion; Shoulder thoracohumeral angles describe the arm position – consistent with the coordinate system used in the musculoskeletal model. The start point represents initial contact of the hand with the pushrim, and end point is the instant when the hand leaves the pushrim

* = Sensitive (coefficient magnitudes > 0.40); ** = Highly sensitive (coefficient magnitudes > 0.75)

The results in Table 2 show several key points. Musculotendon complexes are most sensitive at the beginning of the propulsion cycle (hand contact), with fewer musculotendon complexes showing high sensitivity at the end of the propulsion cycle (hand release). Further, several muscles exhibit significantly higher sensitivity than others, including the posterior and lateral deltoid, teres major, latissimus dorsi, coracobrachialis, and biceps brachii. These results suggest that there is a higher risk of injury during initial hand contact over hand release, and that at the hand contact these key muscles are most sensitive to the angular changes, and thus at risk for injury. The longer muscles overall appear to have lower sensitivity, and hypersensitivity in the shorter musculotendons suggests a higher risk of injury. These results may be limited due to the use of only data from a representative subject for this model analysis. Future work is suggested to confirm these results in a larger population.

### Repeatability analysis

**Fig. 5:**
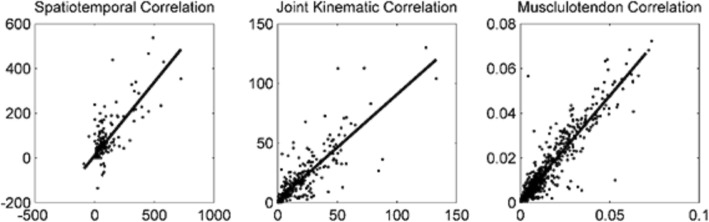
Inter-Trial Pearson Correlation for Categorical Metrics

For each pediatric manual wheelchair propulsion assessment, two separate kinematic trials were recorded for each subject. Statistical correlation analysis was performed to determine inter-trial measurement repeatability of the system, with the results summarized in Table 3. Figure 5 shows that the Pearson correlation coefficients for spatiotemporal parameters, joint range of motion, and musculotendon excursion were high and correlations were significant for all parameters, demonstrating inter-trial measurement repeatability of the system. An additional finding of note is that the metrics with higher Pearson correlation coefficients are the metrics with the least standard deviation in the data, and vice versa. This may suggest that within-subject variability is inversely related to the repeatability of inter-trial measurements.

**Table 3:** Inter-Trial Measurement Repeatability

Metric Type	Pearson Correlation Coefficient	Significance (p)
Spatiotemporal Parameters	0.792	0.001*
Joint Range of Motion	0.853	0.001*
Musculotendon Excursion	0.931	0.001*

Results of correlation analysis. Each individual subject was tested twice under self-selected conditions with no control of speed or power output, and the two measures for each subject are compared

* *p*-value significant at α = 0.05

## Discussion

The system developed in this project uses a combination of consumer-grade hardware and open-source musculoskeletal modeling software to create a unique, cost-effective, efficient, and appropriate analysis technique for clinical research in manual wheelchair biomechanics. The Microsoft Kinect was chosen because of its low cost and ease of use. The OpenSim upper extremity model brings significant computational power to the system, and the interface allowing its use with the Kinect and automating the protocol is the key development of this work. Characterization of the system in several settings has demonstrated its effectiveness for its intended applications. The system adds value to clinical assessments by extracting metrics that other methods, such as standardized outcome tools, cannot.

Comparison of the markerless wheelchair propulsion assessment system against other common outcome measurement protocols reveals several key differences. When compared to laboratory marker-based motion analysis techniques [1] the markerless system requires less space (due to the stationary wheelchair platform), reduced training requirements, and allows faster assessment. However, the marker-based systems have higher precision, and include kinetic assessment and EMG data. Inertial measurement units [4] and instrumented wheels ([11], and [10]) have similar ease of use when compared to the markerless system, and require less time and training to implement than marker-based systems. However, inertial measurement units and instrumented wheels do not provide complete kinematic outputs, but only partial or supplemental data. Inertial measurement units and instrumented wheels are possible future expansion options for the markerless system to permit kinetics to be included in the model. Standardized outcome measures [12] have fewer equipment and technological requirements, but do require trained observation. It is these evaluations that the markerless system is intended to supplement, by adding objective, quantitative outcomes, while adding minimal time and expense.

Manual wheelchair skills capacity is a key therapeutic outcome and can affect quality of life [38]. Assessments using standardized outcome tools in a community setting have documented improvements in response to therapy [39], and shown that levels of physical activity can impact the risk of shoulder pain [40]. Additionally, clinical guidelines suggest monitoring propulsion pattern and technique employed, daily activities, and exercise or therapeutic activities, as a means to assess risk of upper limb pain or injury [41]. This monitoring is important to continuing research in the field, given the paucity of research into therapeutic outcomes of manual wheelchair users [42]. The system proposed here has several key benefits and limitations relative to both standardized outcome tools and complex laboratory motion capture. Clinically, a physical therapist can use the system to assess the UE kinematics and propulsion pattern of wheelchair users as part of routine therapy visits, to track progress. The system provides reliable quantitative data to track patient progress, which is rarely available in current physiotherapeutic clinical assessments. Propulsion pattern, for instance, impacts upper extremity muscle power and stress in manual wheelchair users [43], and is produced by the system. The system enhances the capability of therapists to obtain quantitative data without requiring overly complex and detailed analysis, such as laboratory motion capture [1]. Basic data includes speed, cadence, and propulsion pattern, which are difficult to measure accurately by video or other techniques. The system is effective in this role because it requires minimal training – the software is largely automated and therapists can be trained to use it in a short time.

Several limitations of this work exist and should be addressed. The present system does not include power output, which would require kinetic detection hardware, and is a common metric presented in wheelchair biomechanics literature [37, 44]. The present system also does not measure wheel speed directly, but an algorithm has been implemented to estimate ground speed based on a known wheel diameter and hand motion. Cadence is measured by the system and the resistance level is adjustable based on wheelchair wheel diameter. The lack of speed and power output data reduces the ability to control testing conditions for these metrics, which would be desirable for consistency, such as tracking changes among preferred cadence, wheelchair specifications, and personal differences over time. The addition of speed and power output would increase usability of the system, allowing tracking of changes in preferred cadence, wheelchair styles, and personal performance.

Inter-trial repeatability was significant for spatiotemporal parameters, joint kinematics, and musculotendon excursions. This suggests that the markerless wheelchair propulsion kinematic assessment system is a repeatable measurement tool for pediatric manual wheelchair users, and detects changes that are greater than the inherent normal variability in the population. Given inter-trial repeatability with significant correlation, the system is recommended for further quantitative assessment use in pediatric manual wheelchair users. However, it should be noted that the markerless technology has limitations in precision of kinematic detection, and more advanced technologies may be required to obtain higher precision.

## Conclusions

There is a significant deficit in current literature on manual wheelchair propulsion biomechanics and physiotherapeutic treatment for this population. The system is suggested for immediate implementation in novel research to resolve these key deficiencies in current literature, leading to more effective point-of-care clinical outcome assessments for manual wheelchair users, provided the limitations of markerless technology are taken into account. In the future, home use and telerehabilitation development are suggested as possible directions for the project.
